# Polymorphism analysis of the chloroplast and mitochondrial genomes in soybean

**DOI:** 10.1186/s12870-022-04028-3

**Published:** 2023-01-07

**Authors:** Yanlei Yue, Jiawen Li, Xuegang Sun, Zhen Li, Bingjun Jiang

**Affiliations:** 1 College of Life Sciences, Henan Agricultural University, Zhengzhou, 450002 China; 2 MARA Key Lab of Soybean Biology (Beijing), Institute of Crop Sciences, The Chinese Academy of Agricultural Sciences, Beijing, 100081 China

**Keywords:** Soybean, Mitochondrial genome, Chloroplast genome, Haplotype, mitochondrial heterozygosity

## Abstract

**Background:**

Soybean is an important protein- and oil-rich crop throughout the world. Much attention has been paid to its nuclear genome, which is bi-parentally inherited and associated with many important agronomical traits. However, less is known about the genomes of the semi-autonomous and essential organelles, chloroplasts and mitochondria, of soybean.

**Results:**

Here, through analyzing the polymorphisms of these organelles in 2580 soybean accessions including 107 wild soybeans, we found that the chloroplast genome is more variable than the mitochondrial genome in terms of variant density. Consistent with this, more haplotypes were found in the chloroplast genome (44 haplotypes) than the mitochondrial genome (30 haplotypes). These haplotypes were distributed extremely unevenly with the top two haplotypes (CT1 and CT2 for chloroplasts, MT1 and MT2 for mitochondria) accounting for nearly 70 and 18% of cultivated soybean accessions. Wild soybeans also exhibited more diversity in organelle genomes, harboring 32 chloroplast haplotypes and 19 mitochondrial haplotypes. However, only a small percentage of cultivated soybeans shared cytoplasm with wild soybeans. In particular, the two most frequent types of cytoplasm (CT1/MT1, CT2/MT2) were missing in wild soybeans, indicating that wild soybean cytoplasm has been poorly exploited during breeding. Consistent with the hypothesis that soybean originated in China, we found that China harbors the highest cytoplasmic diversity in the world. The geographical distributions of CT1–CT3 and MT1–MT3 in Northeast China were not significantly different from those in Middle and South China. Two mitochondrial polymorphism sites, p.457333 (T > C) and p.457550 (G > A), were found to be heterozygous in most soybeans, and heterozygosity appeared to be associated with the domestication of cultivated soybeans from wild soybeans, the improvement of landraces to generate elite cultivated soybeans, and the geographic adaptation of soybean.

**Conclusions:**

The haplotypes of thousands of soybean cultivars should be helpful in evaluating the impact of cytoplasm on soybean performance and in breeding cultivars with the desired cytoplasm. Mitochondrial heterozygosity might be related to soybean adaptation, and this hypothesis needs to be further investigated.

**Supplementary Information:**

The online version contains supplementary material available at 10.1186/s12870-022-04028-3.

## Background

Soybean [*Glycine max* (L.) Merr.] is an important crop that is a good source of protein and oil. Because of population growth, it is increasingly required as a source of human food and animal feed throughout the world. However, because of very limited soil resources and government regulations preferring the planting of cultivars that use less water and energy, breeding soybean cultivars with high yield, excellent performance, and low cost requirements is still a great challenge. The development and application of bioinformatics tools have greatly promoted biological research and molecular breeding of soybean. Since the development of second- and third-generation high-throughput sequencing technologies, high-quality reference genomes have been sequenced and assembled for many cultivated soybean varieties including Williams 82 [1] and ZhongHuang 13 [2] and for wild soybean accessions such as W05 [3]. Moreover, various whole-genome resequencing projects have been performed to elucidate the mechanisms underlying the domestication of cultivated soybeans from wild soybean [4], the improvement of landraces to generate elite soybeans [5], and the enhancement of elite soybeans to develop widely planted soybeans [6]. These projects have also identified millions of polymorphic sites. Furthermore, genome-wide association studies and high-density linkage analyses have promoted the dissection of many agronomically important traits [7–9]. These studies have dramatically promoted the breeding of soybeans.

In addition to the nuclear genome, organelle genomes also play an important role in plant growth and development [10, 11]. Specifically, mitochondria are indispensable organelles involved in ATP generation and respiration, and chloroplasts are plant-specific organelles that perform photosynthesis. In contrast to the nuclear genome, which generally follows a bi-parental Mendelian inheritance model, the genomes of these two types of organelles are almost always inherited from the maternal parent [12]. However, due to the complex double fertilization phenomenon in higher plants, the inheritance modes of organelle genomes are also diverse; for example, cucumber is a well-known exception, where the chloroplast is maternally inherited, and the mitochondrial genome is paternally inherited [13]. The chloroplast and mitochondrial genomes of soybean have long been believed to be maternally inherited [14–16].

The study of organelle genomes in rice and corn has facilitated high-yield breeding based on heterosis. As for soybean, due that it is a strict cleistogamous self-pollinator with small flowers, the development and application of heterosis has been hampered by a technical bottleneck. It is time-consuming to emasculate the flowers, making it difficult to generate hybrids of different accessions. It is especially difficult to generate hybrids for distantly related germplasms, which generally exhibit different flowering times. The application of male sterile mutants can partially overcome this difficulty because these mutants have to be cross-pollinated. A recurrent breeding strategy based on a nuclear male sterile mutant, *ms1*, has been used to produce several soybean varieties [17]. However, its breeding efficiency is yet significantly limited due that it is difficult to distinguish the male sterile mutant seeds (*ms1ms1*) from the fertile seeds (*ms1MS1* and *MS1MS1*) before sowing. The utilization of cytoplasmic male sterile (CMS) mutants can effectively solve this problem; Sun et al. (1993) developed the first CMS sterile line OA and maintainer line OB to produce a large number of CMS sterile line, and a CMS-based three-line hybridization breeding system has been successfully established in soybean and used to generate several hybrid cultivars that have been commercially released [15]. However, judging by the fact that these hybrid cultivars have not been widely adopted worldwide, the three-line system has shown limited success. Therefore, it is necessary to elucidate the interactions between mitochondria and nuclei to promote soybean hybrid breeding.

In recent years, there has been some progress in elucidating the molecular biological basis of soybean hybrid breeding. Wang et al. (2021) identified the nuclear gene *GmPRR576* as a fertility restorer gene [18], and He et al. (2021) proposed that the mitochondrial genes *orf178* and *orf261* are CMS-associated candidate genes [19]. Chang et al. (2013) and Liu et al. (2021) de novo assembled the mitochondria genomes of soybean cultivars Aiganhuang [20] and Williams 82 [21], respectively, and revealed the complex structures of these genomes and identified several structural variations. However, the polymorphisms and diversity of soybean organelle genomes are poorly characterized, and the influence of artificial breeding selection on the organelle genome is also poorly understood. Here, to reduce this gap in knowledge, we extracted organelle genome sequences from existing whole-genome resequencing data and performed polymorphism analysis. Our findings shed light on the evolution of organelle genomes and the interaction of the nucleus and cytoplasm and will help promote soybean breeding.

## Results

### Chloroplast and mitochondrial genomes harbor a large number of polymorphisms

**Fig. 1:**
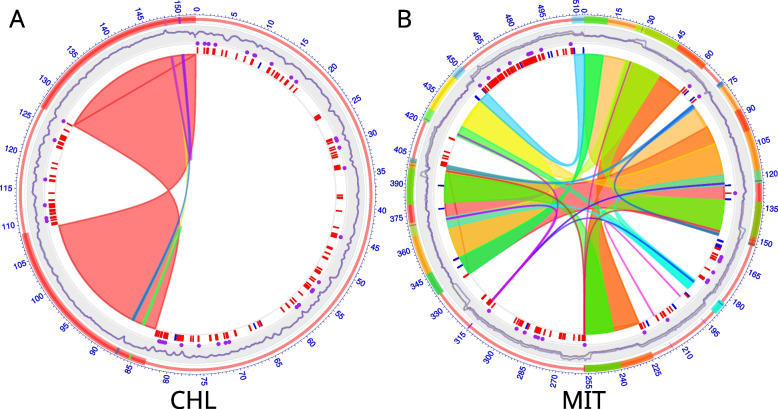
The distribution of polymorphic sites in the soybean chloroplast (CHL) and mitochondrial (MIT) genomes. In the Circos plots of the CHL (**A**) and MIT (**B**) genomes, the paired replicated regions are indicated by bars with the same color in the outer layer and are linked by Bezier curves with the same color. The line in the second layer indicates the sequencing depth. The purple dots in the third layer indicate the tag polymorphic sites used for haplotype analysis, and the red and blue short segments in the fourth layer indicate the SNP and InDel sites, respectively

To identify polymorphisms in organelles, whole-genome resequencing data for 2580 soybean accessions were mapped to the chloroplast and mitochondrial genomes, which contain 152,220 and 513,779 base pairs respectively. After excluding reads mapping to repeat regions, which account for 36.4 and 60.5% of the chloroplast and mitochondrial genomes, respectively (Fig. 1A and B), 182 and 275 high-quality polymorphic sites were called in the chloroplast and mitochondrial genomes, respectively (Table S1). Of the polymorphic sites in chloroplasts, 42 were multi-allelic sites and 140 were bi-allelic sites (Table S1). Of the bi-allelic sites, 113 were SNPs (single nucleotide polymorphisms) and 27 were InDels (inserts and deletions), and 37 SNPs and 3 InDels were located in predicted chloroplast genes (Table S1). In some accessions, polymorphisms caused frameshift and missense mutations in 11 chloroplast genes, namely *atpA*, *ccsA*, *matK*, *ndhF*, *psbB*, *psbM*, *rpoC2*, *rps18*, *rps2*, *rps3*, and *ycf1* (Table S2). Of the polymorphic sites in mitochondria, 25 were multi-allelic sites and 250 were bi-allelic sites (Table S1). Of these bi-allelic sites, 119 were SNPs and 131 were InDels, and 17 SNPs and 2 InDels were located in predicted mitochondrial genes (Table S1). In some accessions, polymorphisms resulted in frameshift and missense changes in seven mitochondrial genes, namely *ccmFc_1*, *cox2_1*, *nad4_1*, *nad5_1*, *rpl5_1*, *rpl16_1*, and *rps3_1* (Table S3). Compared with the chloroplast genomes, mitochondrial genomes contained a higher ratio of InDel sites but a lower ratio of multi-allelic sites. Moreover, the transitions/transversions ratio was 0.2 for the chloroplast genome and 0.99 for the mitochondrial genome. Taken together, these results indicated that the chloroplast and mitochondrial genomes of cultivated and wild soybeans harbor many polymorphisms.

### Thousands of chloroplast genomes can be grouped into 44 haplotypes

**Fig. 2:**
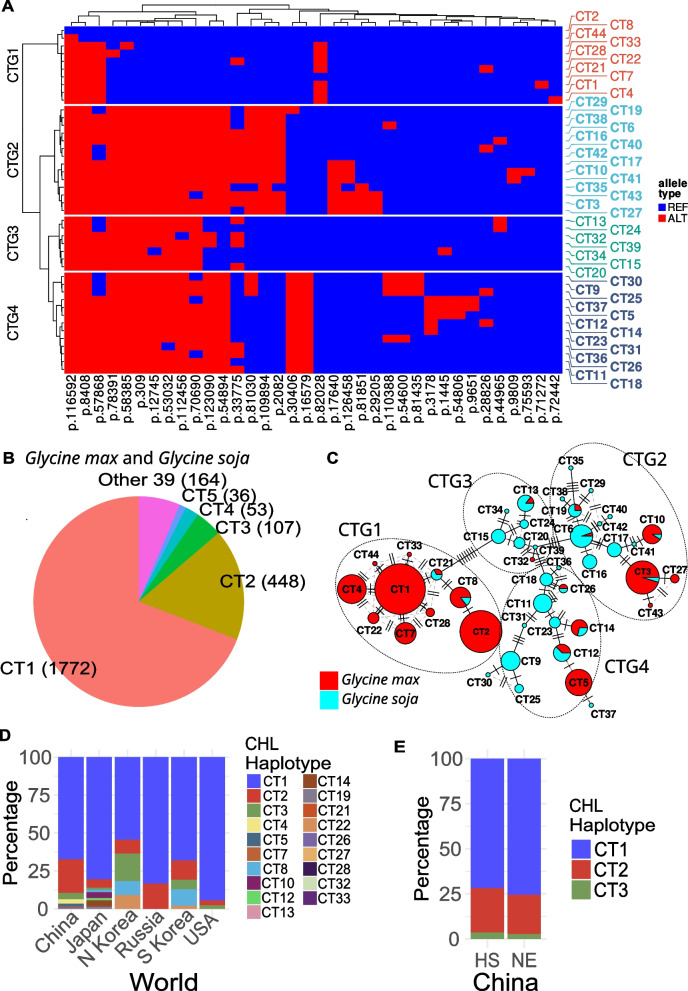
Soybean chloroplast haplotypes and their distribution. **A** Chloroplast haplotypes and tag SNPs. The polymorphic sites are shown below the heat map. The name of each site indicates the position (bp) in the reference chloroplast genome. **B** The overall distribution of chloroplast haplotypes in *Glycine max* and *Glycine soja*. The number in parentheses indicates the number of varieties with the respective haplotype. **C** Network of chloroplast haplotypes in *G. max* and *G. soja*. The number of soybean accessions with each haplotype is indicated by the size of the pie chart. **D** The distributions of soybean chloroplast haplotypes in different countries. **E** The distributions of soybean chloroplast haplotypes in the main production regions of China. CT1–CT44, Chloroplast haplotypes; CTG1–CTG4, Chloroplast haplotype groups; REF, reference alleles; ALT, alternative alleles; CHL, Chloroplast; HS, Huang-Huai-Hai region and South China; NE, Northeast China; N Korea, North Korea; S Korea, South Korea

Based on the linkage between polymorphic sites, 182 chloroplast polymorphisms were represented by 36 tag SNPs. Furthermore, using these tag SNPs the chloroplasts of the 2580 soybean accessions were classified into 44 haplotypes, which were distributed unevenly between cultivated and wild accessions (Fig. 2A, B and Table 1). Only 22 haplotypes were found in the 2473 cultivated soybeans (*G. max*) (Fig. 2C). Of these, CT1 and CT2 were the two most abundant, accounting for 71.7 and 18.1% of the cultivated soybeans, respectively. Although there were only 107 wild soybean lines, which is about 4% of the number of cultivated soybeans, 32 haplotypes were found, which is 10 more than the number found in cultivated soybeans. Of these haplotypes, CT6 and CT9 were the two most abundant, accounting for 16.8 and 10.3% of wild soybeans, respectively. More importantly, the top two haplotypes in cultivated soybeans, CT1 and CT2, were not found in wild soybeans. Similarly, of the top two haplotypes in wild soybeans, CT6 was only found in one cultivated soybean accession and CT9 was not found in any cultivated soybean. About 5.5% (137) of cultivated soybeans shared 10 haplotypes (CT3, CT6, CT8, CT10, CT12, CT13, CT14, CT19, CT21, and CT26) with 45 wild soybeans. Furthermore, based on the difference between haplotypes, the 44 chloroplast haplotypes were clustered into 4 groups (CTG1–CTG4, Fig. 2 and Table 1). CTG1 and CTG2 were the two dominant groups containing the dominant haplotyes CT1 and CT3, respectively. CTG1 mainly consisted of cultivated soybean haplotypes, while CTG2, CTG3, and CTG4 were mainly composed of wild soybean haplotypes.

**Table 1:** Chloroplast haplotypes and haplotype groups

Haplotype group	Haplotype	Haplotype group	Haplotype	Haplotype group	Haplotype	Haplotype group	Haplotype
CTG1	CT1	CTG2	CT3	CTG3	CT13	CTG4	CT5
	CT2		CT6		CT15		CT9
	CT4		CT10		CT20		CT11
	CT7		CT16		CT24		CT12
	CT8		CT17		CT32		CT14
	CT21		CT19		CT34		CT18
	CT22		CT27		CT39		CT23
	CT28		CT29				CT25
	CT33		CT35				CT26
	CT44		CT38				CT30
			CT40				CT31
			CT41				CT36
			CT42				CT37
			CT43				

### Thousands of mitochondrial genomes can be grouped into 30 haplotypes

**Fig. 3:**
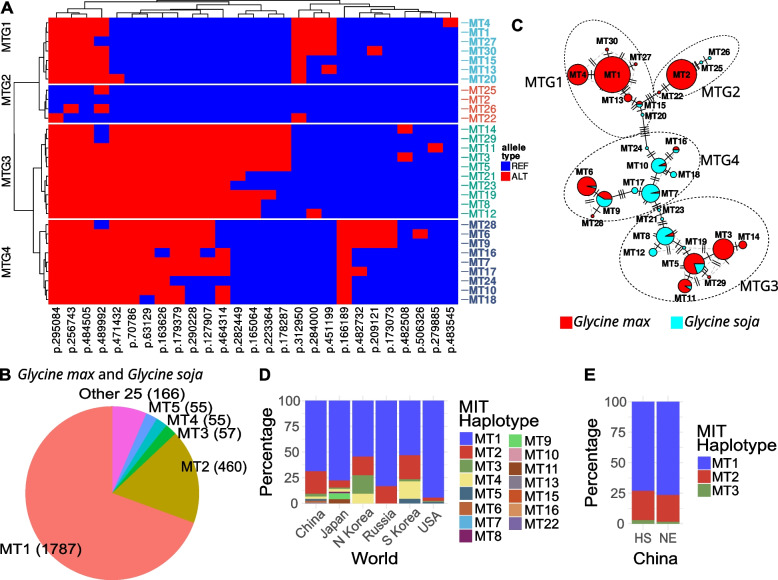
Soybean mitochondrial haplotypes and their distribution. **A** Mitochondrial haplotypes and tag SNPs. The polymorphic sites are shown below the heat map. The name of each site indicates the position (bp) in the reference mitochondrial genome. **B** The overall distribution of mitochondrial haplotypes in *Glycine max* and *Glycine soja*. The number in parentheses indicates the number of varieties with the respective haplotype. **C** Network of mitochondrial haplotypes in *G. max* and *G. soja*. The number of soybean accessions with each haplotype is indicated by the size of the pie chart. **D** The distributions of soybean mitochondrial haplotypes in different countries. **E** The distributions of soybean mitochondrial haplotypes in the main production regions of China. MT1–MT30, mitochondrial haplotypes; MTG1–MTG4, mitochondrial haplotype groups; REF, reference alleles; ALT, alternative alleles; MIT, mitochondria; HS, Huang-Huai-Hai region and South China; NE, Northeast China; N Korea, North Korea; S Korea, South Korea

Based on the linkage between polymorphic sites, 275 mitochondrial polymorphic sites were represented by 27 tag SNPs. In total, 30 mitochondrial haplotypes were found in the 2580 soybean accessions (Fig. 3A and Table 2). Like the chloroplast haplotypes, these mitochondrial haplotypes were also distributed unevenly between wild and cultivated accessions (Fig. 3B). Twenty haplotypes were found in cultivated soybeans (Fig. 3C). Of these, MT1 and MT2 were the two most abundant, accounting for 72.3 and 18.6% of cultivated soybeans, respectively. In contrast, 19 haplotypes were found in wild soybeans. Of these, MT7 and MT8 were the two most abundant, accounting for 27.1 and 23.4% of wild soybeans, respectively. As was the case for the chloroplast genomes, the dominant haplotypes in cultivated soybeans, MT1 and MT2, were not found in wild soybeans. Similarly, the dominant haplotypes in wild soybeans, MT7 and MT8, were only found in one and two cultivated soybeans respectively. About 4.2% (103) of cultivated soybeans shared 9 haplotypes (MT5, MT6, MT7, MT8, MT9, MT10, MT11, MT15, and MT16) with 93 wild soybeans. The 30 mitochondrial haplotypes were clustered into 4 groups (MTG1–MTG4, Fig. 3 and Table 2). MTG1, which contained MT1, and MTG2, which contained MT2, were the two dominant groups. MTG1 was mainly composed of cultivated soybean haplotypes, while MTG4 was mainly composed of wild soybean haplotypes.

**Table 2:** Mitochondrial haplotypes and haplotype groups

Haplotype group	Haplotype	Haplotype group	Haplotype	Haplotype group	Haplotype	Haplotype group	Haplotype
MTG1	MT1	MTG2	MT2	MTG3	MT3	MTG4	MT6
	MT4		MT22		MT5		MT7
	MT13		MT25		MT8		MT9
	MT15		MT26		MT11		MT10
	MT20				MT12		MT16
	MT27				MT14		MT17
	MT30				MT19		MT18
					MT21		MT24
					MT23		MT28
					MT29		

### The geographical distribution of chloroplast and mitochondrial haplotypes

Consistent with the uneven distribution of chloroplast and mitochondrial haplotypes, the two most frequent haplotypes overall, i.e., CT1 and CT2 for chloroplasts and MT1 and MT2 for mitochondria, were also the top two haplotypes in all countries investigated except North Korea, where CT1 and CT3 were the top two chloroplast haplotypes (Figs. 2D and 3D). In China, 16 chloroplast haplotypes and 13 mitochondrial haplotypes were found in cultivated soybeans, and the top three haplotypes, i.e., CT1, CT2, and CT3 for chloroplasts, and MT1, MT2, and MT3 for mitochondria, were distributed similarly in Northeast China and in the Huang-Huai-Hai region and South China (Figs. 2E and 3E).

**Fig. 4:**
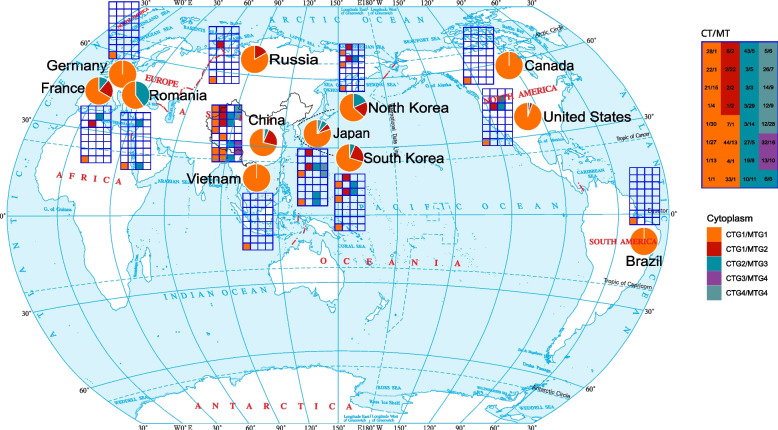
Distribution of soybean cytoplasm groups in the world. Pie chart indicates the composition of cytoplasm groups in a country. Tile chart indicates the composition of cytoplasm groups in a country. In the CT/MT tile chart (upper right), the numbers indicate the CT and MT haplotypes (e.g., 1/1 indicates CT1/MT1). The cytoplasm types in the CT/MT table with the same color belong to the same cytoplasm group

**Fig. 5:**
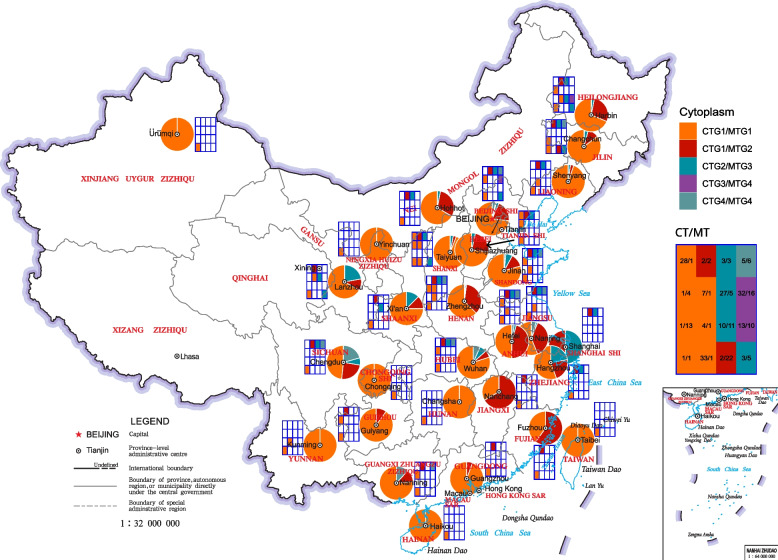
Distribution of soybean cytoplasm groups in China. Pie chart indicates the composition of cytoplasm groups in a province. Tile chart indicates the composition of cytoplasm groups in a country. In the CT/MT tile chart (upper right), the numbers indicate the CT and MT haplotypes (e.g., 1/1 means the cytoplasm is CT1/MT1). The cytoplasm groups in the CT/MT table with the same color belong to the same cytoplasm group

Among cultivated soybean accessions, 1712 (69.2%) harbored the CT1 haplotype and the MT1 haplotype and are referred to as CT1/MT1; 447 (18.1%) harbored CT2/MT2; and 57 (2.3%) harbored CT3/MT3. When looking at haplotype groups, 1848 (74.7%), 461 (18.6%), and 117 (4.7%) accessions harbored CTG1/MTG1, CTG1/MTG2, and CTG2/MTG3 respectively. These haplotype groups were distributed unevenly in different countries (Fig. 4) and in different provinces in China (Fig. 5). Moreover, China, Japan, South Korea, and North Korea harbored the highest diversity of cytoplasm. In China, the provinces located in North China and Northeast China harbored the highest diversity.

### Two heterozygous mitochondrial polymorphism sites might be associated with soybean breeding

**Fig. 6:**
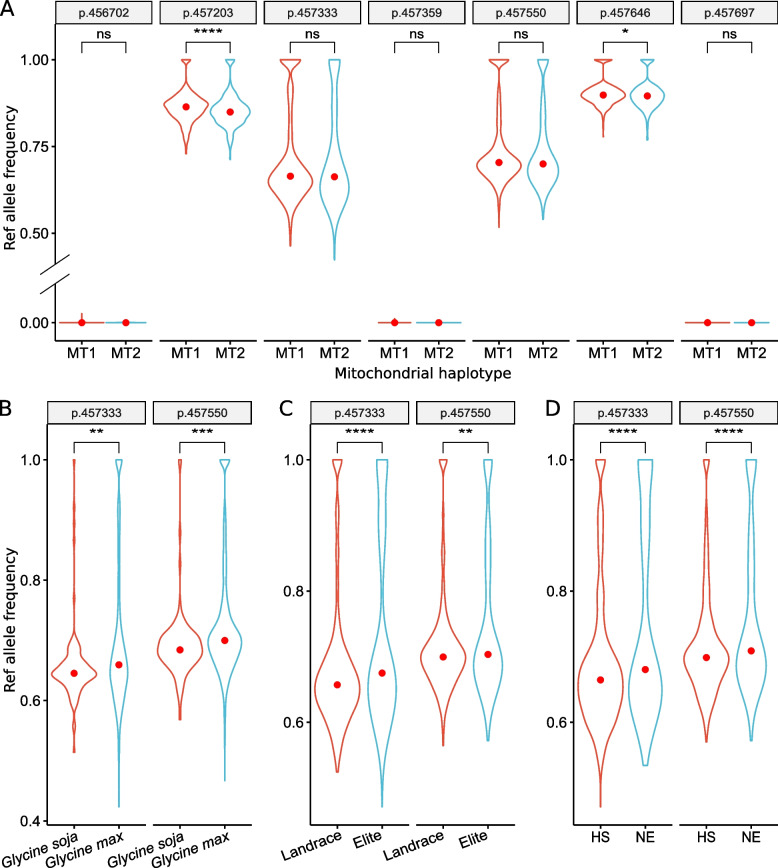
Two heterozygous mitochondrial polymorphism sites, p.457333 (T > C) and p.457550 (G > A), might be associated with soybean adaptation. **A-D** The reference allele frequencies of two heterozygous mitochondrial polymorphism sites and the nearby sites in soybean accessions with the mitochondrial haplotype MT1 (red) or MT2 (blue) (**A**), in wild (red) and cultivated (blue) soybeans (**B**) in landraces (red) and elite (blue) cultivated soybeans (**C**), and in cultivated soybeans of Northeast China (red) and in ones from Middle and South China (Huang-Huai-Hai region and South China) (blue) (**D**). NE, Northeast China. HS, Huang-Huai-Hai region and South China

When exploring the heterozygosity of the chloroplast and mitochondria genomes, two mitochondrial polymorphism sites, p.457333 (T > C) and p.457550 (G > A), were found to be heterozygous in most soybeans (Fig. 6A). Specifically, in MT1 and MT2 cultivated soybeans, the median reference allele frequencies (RAFs) of p.457333 were 0.664 and 0.662, respectively, and the median RAFs of p.457550 were 0.704 and 0.700, respectively. However, the median RAFs of the nearby polymorphism sites p.456702 (C > CT), p.457359 (GA > G), and p.457697 (AC > A) were all zero, indicating that these sites were homozygous for alternative alleles. Nearly all of the p.457203 (A > G) and p.456746 (CAGACGA>C) sites were homozygous for the reference alleles. These results showed that the heterozygosity of these two sites was not related to the mitochondrial haplotype. Moreover, the median RAFs were significantly higher in cultivated soybeans than in wild soybeans (Fig. 6B), indicating that heterozygosity might be related to soybean domestication. Similar observations were also made for soybean improvement from landraces to elite cultivated soybeans (Fig. 6C), where elite cultivated soybeans exhibited a significantly higher RAF than landraces. Cultivated soybeans of Northeast China also exhibited a significantly higher RAF than those from Middle and South China (Huang-Huai-Hai region and South China) (Fig. 6D). These results suggested that the mitochondrial heterozygosity might be related to soybean adaptation, a hypothesis that needs to be further investigated.

## Discussion

Chloroplasts and mitochondria are both semi-autonomous organelles that contain relatively independent genomes [12]. RNA transcription, protein translation, and self-replication in these organelles depend on genetic information encoded by the nuclear genome. Although the information encoded by organelle genomes is very limited, these genomes affect plant fertility, photosynthesis, growth, and development [10, 11]. At present, little is known about the structure and function of soybean organelle genomes, which seriously limits the study and application of soybean three-line hybridization. In this study, 2580 soybean accessions (including 107 wild soybeans) were selected to analyze the polymorphisms and haplotypes in organelle genomes using published whole-genome sequencing data. The experimental materials in this study included representative varieties from all over the world and a large number of wild materials, which enabled the comprehensive analysis of the genetic and variation characteristics of soybean mitochondrial and chloroplast genes. The results lay a solid foundation for molecular biology, genetics, and cytology studies of soybean organelles, and provide a theoretical basis for the study and application of soybean heterosis.

Mitochondrial genomes are specific to each plant species; they have variable sizes, complex structures, and different patterns of gene loss and gain during evolution [22]. The higher complexity compared with the nuclear and chloroplast genomes makes the study of soybean mitochondrial genomes more difficult. Although biologists have studied the soybean mitochondrial genome for more than 30 years, its sequence is still ambiguous because it has a complex structure with a large number of repeat regions. However, the chloroplast genome is smaller in size, stabler in structure, and more highly conserved in sequence compared with the mitochondrial genome [23]. It is therefore more suitable for pedigree analysis and evolutionary studies aimed at tracing maternal sources. Thus, it is essential to explore the diversity of organelle genomes and the characteristics of variation in soybeans to address production problems that cannot be easily solved at the level of the nuclear genome such as cytoplasmic fertility and photosynthesis. In our study, we found that although the mitochondria and chloroplast genomes are relatively more conserved than nuclear genomes, they also harbor many polymorphic sites. Moreover, some chloroplast and mitochondria genes contain missense or frameshift mutations in some accessions; examples include genes related to ATP synthesis such as *atpA*, *ccsA*, *matK*, *ndhF*, *psbB*, *psbM*, *rpoC2*, *rps18*, *rps2*, and *rps3*, and genes related to photosynthesis such as *ycf1*, *ccmFc_1*, *cox2_1*, *nad4_1*, *nad5_1*, *rpl5_1*, *rpl16_1*, and *rps3_1*, which are target genes for improving the efficiency of the mitochondria and chloroplast.

The number of polymorphic loci directly reflects the variability of species and reflects the direction of natural and artificial selection. In this study, we found that the chloroplast and mitochondrial genomes of soybean harbor different numbers of polymorphic sites. However, chloroplast genomes showed a higher level of nucleotide diversity than mitochondrial genomes (Table S1). Moreover, chloroplast genomes had more multi-allelic polymorphic sites, while the mitochondrial genomes showed a high number of InDels, indicating that mitochondria experience greater selection pressure and that both organelles have different mechanisms to maintain the conservation of genomes. We also found that cultivated and wild soybeans have different haplotypes in both the chloroplast and mitochondria genomes. Although the top two haplotypes of chloroplast and mitochondria accounted for nearly 90% of cultivated soybeans, they were not found in wild soybeans. Only a small percentage of cultivated soybeans had the same haplotypes as wild soybeans. These results indicated that cultivated soybeans have diverged from wild soybeans since their domestication, and that there have been few exchanges of genetic information between cultivated soybeans and wild soybeans, or that wild soybeans are usually used as the paternal parent in hybridization crosses. Moreover, the highest number of haplotypes was found in China, which supports the notion that soybean originated in China. This is consistent with the conclusion about the origin of soybean from the analysis of soybean genome evolution [24, 25]. However, the organelle haplotypes showed similar distributions in the three main production regions of China, Northeast China, the Huang-Huai-Hai region, and South China. This indicates that CT1/MT1 cytoplasm might be the original cytoplasm because cytoplasm was rarely selected until the CMS phenomenon was discovered several decades ago.

Interestingly, mitochondria were found to be heterozygous at two polymorphism sites, p.457333 (T > C) and p.457550 (G > A), in most cultivated and wild soybeans (Fig. 6). Although these sites were homozygous for the reference alleles in some soybeans, they were rarely homozygous for the alternative alleles. The mitochondrial heterozygosity might be associated with the domestication of cultivated soybean from wild soybean, improvement from landraces to elite cultivated soybeans, and the geographic adaptation of soybean (Fig. 6). However, the mechanism leading to the heterozygosity of these two sites needs to be further investigated.

The haplotypes that we have characterized will aid efforts to identify organelle haplotypes, construct a genetic population by hybridizing soybeans with different chloroplast/mitochondria haplotypes, elucidate nucleus-organelle interactions in a population, and finally to improve the efficiency of organelles in terms of energy use and application in soybean breeding.

## Materials and methods

Four publicly available soybean whole-genome resequencing projects were downloaded from public databases. PRJNA291452 [6], PRJNA589345 [26], and PRJNA257011 [5] were downloaded from the NCBI Sequence Read Archive database (https://www.ncbi.nlm.nih.gov). CRA002269 [27] was downloaded from the Genome Sequence Archive [28] in the National Genomics Data Center [29], China National Center for Bioinformation/Beijing Institute of Genomics, Chinese Academy of Sciences (https://ngdc.cncb.ac.cn/). A total of 2580 soybean accessions were used (Table S4), 2473 of which were cultivated soybeans and 107 were wild soybeans. The accessions came from China (1717), the United States (180), Japan (72), South Korea (47), Russia (42), North Korea (11), Canada (9), France (9), Germany (5), Romania (5), Brazil (4), Vietnam (4), the Netherlands (3), former Serbia and Montenegro (2), Georgia (2), Belgium (1), Denmark (1), Peru (1), Sweden (1), and Ukraine (1), and 463 accessions were of unknown origin.

The downloaded raw reads were first quality-trimmed with TRIMMOMATIC (parameter: ILLUMINACLIP:TruSeq3-PE.fa:2:30:10 LEADING:3 TRAILING:3 SLIDINGWINDOW:4:15 MINLEN: 75) [30], then mapped to the reference genome of the famous Chinese variety ZhongHuang 13 using BWA-mem with default parameters [2, 31]. The properly paired reads that uniquely mapped to the chloroplast and mitochondrial genomes without alternative alignments, soft clips and hard clips were selected for further analysis through the filter command in the BamTools Toolkit (https://github.com/pezmaster31/bamtools). Variants including SNPs and InDels were called using the Genome Analysis Toolkit HaplotypeCaller with default parameters (−stand_call_conf set to 30.0, −stand_emit_conf set to 10.0, and -glm set to BOTH) [32]. High quality variants were obtained after removing the low-quality variants located in duplicated regions of the chloroplast and mitochondrial genomes. The duplicated regions were identified by blastn (−evalue 1e-50) [33]. Annotation of the cytoplasmic variants was carried out using the SnpEff tool [34]. The variants were displayed using the R package OmicCircos [35]. Haplotype analysis was performed using the R package pegas [36], and the results were plotted using the R packages ggplot2 and ComplexHeatmap [37, 38].

## Supplementary Information

**Additional file 1: Table S1.** Basic statistics for the polymorphic sites in the soybean chloroplast and mitochondrial genomes.

**Additional file 2: Table S2.** Polymorphic sites with a high or moderate impact in chloroplast genomes.

**Additional file 3: Table S3.** Polymorphic sites with a high or moderate impact in mitochondrial genomes.

**Additional file 4: Table S4.** Chloroplast and mitochondrial haplotype in soybean varieties.

## Data Availability

The datasets generated and/or analyzed during the current study are available in the NCBI Sequence Read Archive database (https://www.ncbi.nlm.nih.gov, PRJNA291452 [6], PRJNA589345 [26], and PRJNA257011 [5]) and in the National Genomics Data Center [29], China National Center for Bioinformation/Beijing Institute of Genomics, Chinese Academy of Sciences (https://ngdc.cncb.ac.cn, CRA002269 [27]).
