# Supplementary material for: Polymorphism analysis of the chloroplast and mitochondrial genomes in soybean
Source: BMC Plant Biol. 2023 Jan 7;23:15. doi: 10.1186/s12870-022-04028-3 (PMC9825035; doi:10.1186/s12870-022-04028-3)
Supplement: Supplementary file 1 — Additional file 1: Table S1. Basic statistics for the polymorphic sites in the soybean chloroplast and mitochondrial genomes. [file 12870_2022_4028_MOESM1_ESM.docx]

**[Table S1. Basic statistics for the polymorphic sites in](javascript:void(0);) the [soybean chloroplast and mitochondrial genomes.](javascript:void(0);)**

|  | [Chloroplast genome](javascript:void(0);) | [Mitochondrial genome](javascript:void(0);) |
| --- | --- | --- |
| [Reference size (bp)](javascript:void(0);) | [152220](javascript:void(0);) | [513779](javascript:void(0);) |
| [total sites](javascript:void(0);) | [182](javascript:void(0);) | [275](javascript:void(0);) |
| [multi-allele sites](javascript:void(0);) | [42](javascript:void(0);) | [25](javascript:void(0);) |
| [bi-allele sites](javascript:void(0);) | [140](javascript:void(0);) | [250](javascript:void(0);) |
| [--SNP number](javascript:void(0);) | [113](javascript:void(0);) | [119](javascript:void(0);) |
| [---- ts/tv](javascript:void(0);) | [0.2](javascript:void(0);) | [0.99](javascript:void(0);) |
| [---- HIGH impact](javascript:void(0);) | [0](javascript:void(0);) | [0](javascript:void(0);) |
| [---- MODERATE impact](javascript:void(0);) | [21](javascript:void(0);) | [10](javascript:void(0);) |
| [---- LOW impact](javascript:void(0);) | [16](javascript:void(0);) | [7](javascript:void(0);) |
| [-- Indel number](javascript:void(0);) | [27](javascript:void(0);) | [131](javascript:void(0);) |
| [---- HIGH impact](javascript:void(0);) | [3](javascript:void(0);) | [2](javascript:void(0);) |
| ‍θ_π_ | 1.73e-05 | 1.46e-05 |
