# Supplementary material for: Polymorphism analysis of the chloroplast and mitochondrial genomes in soybean
Source: BMC Plant Biol. 2023 Jan 7;23:15. doi: 10.1186/s12870-022-04028-3 (PMC9825035; doi:10.1186/s12870-022-04028-3)
Supplement: Supplementary file 2 — Additional file 2: Table S2. Polymorphic sites with a high or moderate impact in chloroplast genomes. [file 12870_2022_4028_MOESM2_ESM.docx]

**[Table](javascript:void(0);) S2[.](javascript:void(0);) Polymorphic sites with a high or moderate impact in [chloroplast genomes.](javascript:void(0);)**

| Position | Ref allele | Alt allele | Annotation | Annotation impact | Gene name | Gene id | HGVS.p |
| --- | --- | --- | --- | --- | --- | --- | --- |
| 2082 | G | T | missense | MODERATE | matK | SoyZH13_CG000300 | p.Gln483Lys |
| 2942 | G | T | missense | MODERATE | matK | SoyZH13_CG000300 | p.Ser196Tyr |
| 31361 | A | ACTTAT | frameshift | HIGH | psbM | SoyZH13_CG003100 | p.Ile25fs |
| 40811 | T | G | missense | MODERATE | rpoC2 | SoyZH13_CG003600 | p.Phe80Leu |
| 44678 | CT | C | frameshift | HIGH | rpoC2 | SoyZH13_CG003600 | p.Phe1372fs |
| 44678 | CT | AT | missense | MODERATE | rpoC2 | SoyZH13_CG003600 | p.Phe1369Leu |
| 45229 | CA | C | frameshift | HIGH | rps2 | SoyZH13_CG003700 | p.Ala80fs |
| 51068 | G | T | missense | MODERATE | atpA | SoyZH13_CG004100 | p.Ala400Ser |
| 66710 | G | GA | frameshift | HIGH | rps18 | SoyZH13_CG006300 | p.Tyr101fs |
| 71272 | A | G | missense | MODERATE | psbB | SoyZH13_CG006700 | p.Ile85Met |
| 82358 | A | T | missense | MODERATE | rps3 | SoyZH13_CG007900 | p.Phe83Leu |
| 109164 | C | A | missense | MODERATE | ycf1 | SoyZH13_CG009700 | p.Phe297Leu |
| 109405 | A | C | missense | MODERATE | ycf1 | SoyZH13_CG009700 | p.Lys378Gln |
| 109894 | G | A | missense | MODERATE | ycf1 | SoyZH13_CG009700 | p.Ala541Thr |
| 110239 | GA | G | frameshift | HIGH | ycf1 | SoyZH13_CG009700 | p.Asn660fs |
| 110239 | GA | GAA | frameshift | HIGH | ycf1 | SoyZH13_CG009700 | p.Asn660fs |
| 110388 | G | T | missense | MODERATE | ycf1 | SoyZH13_CG009700 | p.Leu705Phe |
| 110397 | T | G | missense | MODERATE | ycf1 | SoyZH13_CG009700 | p.Ile708Met |
| 110904 | T | G | missense | MODERATE | ycf1 | SoyZH13_CG009700 | p.Asn877Lys |
| 111041 | TA | T | frameshift | HIGH | ycf1 | SoyZH13_CG009700 | p.Thr927fs |
| 111041 | TA | TAA | frameshift | HIGH | ycf1 | SoyZH13_CG009700 | p.Thr927fs |
| 111372 | GA | G | frameshift | HIGH | ycf1 | SoyZH13_CG009700 | p.Ile1038fs |
| 111372 | GA | GAA | frameshift | HIGH | ycf1 | SoyZH13_CG009700 | p.Ile1038fs |
| 111585 | A | T | missense | MODERATE | ycf1 | SoyZH13_CG009700 | p.Leu1104Phe |
| 112022 | T | G | missense | MODERATE | ycf1 | SoyZH13_CG009700 | p.Ile1250Arg |
| 112220 | TA | T | frameshift | HIGH | ycf1 | SoyZH13_CG009700 | p.Ile1320fs |
| 112220 | TA | TAA | frameshift | HIGH | ycf1 | SoyZH13_CG009700 | p.Ile1320fs |
| 112386 | G | T | missense | MODERATE | ycf1 | SoyZH13_CG009700 | p.Leu1371Phe |
| 112456 | A | C | missense | MODERATE | ycf1 | SoyZH13_CG009700 | p.Met1395Leu |
| 113212 | T | G | missense | MODERATE | ycf1 | SoyZH13_CG009700 | p.Tyr1647Asp |
| 113258 | A | C | missense | MODERATE | ycf1 | SoyZH13_CG009700 | p.Tyr1662Ser |
| 122821 | A | C | missense | MODERATE | ccsA | SoyZH13_CG010600 | p.Tyr181Asp |
| 123090 | C | T | missense | MODERATE | ccsA | SoyZH13_CG010600 | p.Cys91Tyr |
| 126458 | A | C | missense | MODERATE | ndhF | SoyZH13_CG011000 | p.Lys689Gln |
