# Supplementary material for: Polymorphism analysis of the chloroplast and mitochondrial genomes in soybean
Source: BMC Plant Biol. 2023 Jan 7;23:15. doi: 10.1186/s12870-022-04028-3 (PMC9825035; doi:10.1186/s12870-022-04028-3)
Supplement: Supplementary file 3 — Additional file 3: Table S3. Polymorphic sites with a high or moderate impact in mitochondrial genomes. [file 12870_2022_4028_MOESM3_ESM.docx]

**[Table](javascript:void(0);) S3[.](javascript:void(0);) Polymorphic sites with a high or moderate impact in [mitochondrial genomes.](javascript:void(0);)**

| Position | Ref allele | Alt allele | Annotation | Annotation impact | Gene name | Gene id | HGVS.p |
| --- | --- | --- | --- | --- | --- | --- | --- |
| 70786 | G | T | missense | MODERATE | nad5_1 | SoyZH13_MG000800 | p.Pro484Gln |
| 210430 | A | T | missense | MODERATE | rpl5_1 | SoyZH13_MG002500 | p.Ile122Leu |
| 210445 | A | T | missense | MODERATE | rpl5_1 | SoyZH13_MG002500 | p.Ile127Phe |
| 270193 | G | T | missense | MODERATE | rpl16_1 | SoyZH13_MG002800 | p.Ala153Asp |
| 270575 | CT | C | frameshift | HIGH | rpl16_1 | SoyZH13_MG002800 | p.Asp27fs |
| 270575 | CT | C | frameshift | HIGH | rps3_1 | SoyZH13_MG002900 | p.Arg552fs |
| 270576 | T | C | missense | MODERATE | rps3_1 | SoyZH13_MG002900 | p.Arg552Gly |
| 270998 | A | C | missense | MODERATE | rps3_1 | SoyZH13_MG002900 | p.Ile411Arg |
| 315078 | G | T | missense | MODERATE | cox2_1 | SoyZH13_MG003600 | p.Ser38Tyr |
| 403564 | TTA | T | frameshift | HIGH | ccmFc_1 | SoyZH13_MG004100 | p.Ile308fs |
| 481170 | C | T | missense | MODERATE | nad4_1 | SoyZH13_MG004800 | p.Asp444Asn |
| 481190 | A | G | missense | MODERATE | nad4_1 | SoyZH13_MG004800 | p.Val437Ala |
| 481305 | C | T | missense | MODERATE | nad4_1 | SoyZH13_MG004800 | p.Gly399Arg |
| 481367 | G | A | missense | MODERATE | nad4_1 | SoyZH13_MG004800 | p.Ser378Phe |
